# Violent Pain—Its Effects on Animals and Men

**Published:** 1877-01

**Authors:** 


Violent Pain—Its Effect on Animals and Men.—Mantegazza (Hirsch Jahresbericht) by experiments proved that in animals violent pain produced loss of appetite, dyspepsia, nausea, arrest of the digestive process, vomiting and diarrhœa, and if continued, great weakness and considerable loss of weight, and greater sensitiveness to all injurious agencies. In man we find that severe neuralgic pain produces dyspepsia, want of appetite, coated tongue, pallor and anæmia, weak pulse, cold extremities, depressed, excitable states of the system, and great sensitiveness to external injurious influences. *Oregon Medical Journal.*

